# Lessons Learned from the Implementation of Youth Wellness Hubs Ontario, an Integrated Youth Services Network: Perspectives from Network Leads

**DOI:** 10.5334/ijic.7605

**Published:** 2024-04-10

**Authors:** Nirupa Varatharasan, Debbie Chiodo, Mary Hanna, Jo Lyn Henderson

**Affiliations:** 1 Youth Wellness Hubs Ontario, CA; 2 Centre for Addiction and Mental Health, CA; 3 Western University, CA; 4 University of Toronto, CA

**Keywords:** Integrated Youth Services, youth mental health, facilitators, barriers, developmental evaluation

## Abstract

**Introduction:**

Mental health and substance use services for youth in Canada continue to be fragmented. In response, Integrated Youth Services (IYS) has been proposed to address gaps in youth mental health services that can lead to improved youth outcomes. Youth Wellness Hubs Ontario (YWHO) was launched in 2017 as Ontario’s IYS Network for youth ages 12–25, prioritizing continuous improvement through evaluation.

**Description:**

At the end of the first three years of the YWHO initiative, an evaluation was carried out to identify the barriers and facilitators to the initial implementation of YWHO and service delivery modifications resulting from the COVID-19 pandemic across ten sites. Reporting on these is the focus of this article. Key informant interviews were conducted in early 2021 with Network Leads from all ten initial YWHO sites. Reflexive thematic analysis was used to analyze all interview data.

**Discussion:**

Facilitators to the implementation of the YWHO model included diversified funding models, YWHO Provincial Office implementation supports, clear hub processes, robust community partnerships, organizational support and dedicated staff. Common barriers included certain challenges related to staffing and finances, implementation of the shared data collection platform, implementation of measurement-based care, partnerships, integrated service delivery, and branding and communications.

**Conclusion:**

Implementation of IYS is highly collaborative and quite complex. As interest in such models increase, so does the need for knowledge related to optimal implementation. Learnings have informed developments and improvements made to the YWHO model. Insights will also inform how stakeholders support youth in their communities in designing and implementing services that improve youth mental health and overall well-being.

## Introduction

### Background

The last decade has seen increased international attention in addressing the unmet mental health and substance use needs of young people. It is well established that adolescence and early adulthood are critical times for the onset of mental health difficulties [1]. Globally, approximately 10–20% of children and adolescents experience mental health disorders [2,3], with 75% of lifetime adult mental health disorders developing during adolescence [1]. In Canada, it is estimated that 1 in 5 youth (12–25 years) experience mental health and substance use disorders [4,5], and suicide is the second leading cause of death in Canadian youth ages 15 to 24 [6]. The COVID-19 pandemic has further exacerbated mental health challenges experienced by youth [7]. Despite increasing need, access to mental health and substance use services is limited; the Canadian Institute for Health Information’s (CIHI) newest data shows that in 2023, three out of five children and youth (age 13 to 24) with self-reported early needs accessed mental health and substance use services. Among young Canadians who accessed mental health services during the last 6 months, about half said they were not easy to access with the top five barriers being timing/wait times (74%), feeling overwhelmed (70%), limited choices including appointment hours (57%), stigma (52%), and being misunderstood/dismissed (50%) [8].

### Problem Statement

In Canada and across the globe, there is growing concern of the barriers to the youth mental health and substance use services systems [9,10,11,12]. Barriers such as system fragmentation, access, complicated pathways to care, lack of developmentally-appropriate and evidence-based services, lack of culturally appropriate services, discontinuation of service at transition to adulthood, and lack of meaningful youth and family engagement, create a need for urgent system transformation [9,10,12,13].

Internationally, Integrated Youth Services (IYS) is seen as a solution to address the critical gaps in the youth mental health and substance use system [10,11,12]. IYS is defined as a collaborative approach to care that brings traditionally separate services together (e.g., mental health, substance use, primary health care, housing, education, etc.) into one community-based setting to provide comprehensive services for youth across adolescent and young adulthood (i.e., ages 12–25 years) and their families. IYS models have been established in Canada (e.g., ACCESS Open Minds, Aire Ouverte, Foundry and Youth Wellness Hubs Ontario (YWHO)) and internationally (e.g., headspace (AU), Jigsaw (IRE), emphasizing timely access, youth friendliness, and holistic care, and integrated services for mental and physical health, substance use, education, employment, peer support, and navigation.

While recent reviews have described the key attributes and emerging evidence from integrated, community-based youth services [10,11,14], there is general agreement that implementing these services is complex and multi-faceted, as it can happen at different levels and through different mechanisms [11,15].

Although models of IYS differ, in almost all cases, integration within community-based youth services has required significant reform that includes co-location of services, integrated governance, shared funding models, service planning and delivery, and technological infrastructure that allows the sharing of youths’ needs across multiple providers. When executed well, integrated care holds significant potential for improving youth outcomes, access to services, and cost and efficiency within the youth service system [11,14]. There is a paucity of peer-reviewed evidence pertaining to the barriers and facilitators of IYS in Canada for youth across adolescent and young adulthood (i.e., ages 12 to 25 years) but notably, there is a recent addition by Chiodo et al., [16] on the barriers and facilitators to a newly established IYS network in a rural community in Ontario. In contrast, the current paper aims to understand the barriers and facilitators of the initial implementation of a large network of IYS in Ontario, Canada called Youth Wellness Hubs Ontario (YWHO) from the perspective of Network Leads.

### Youth Wellness Hubs Ontario: Integrated Youth Services Network

**Figure 1:**
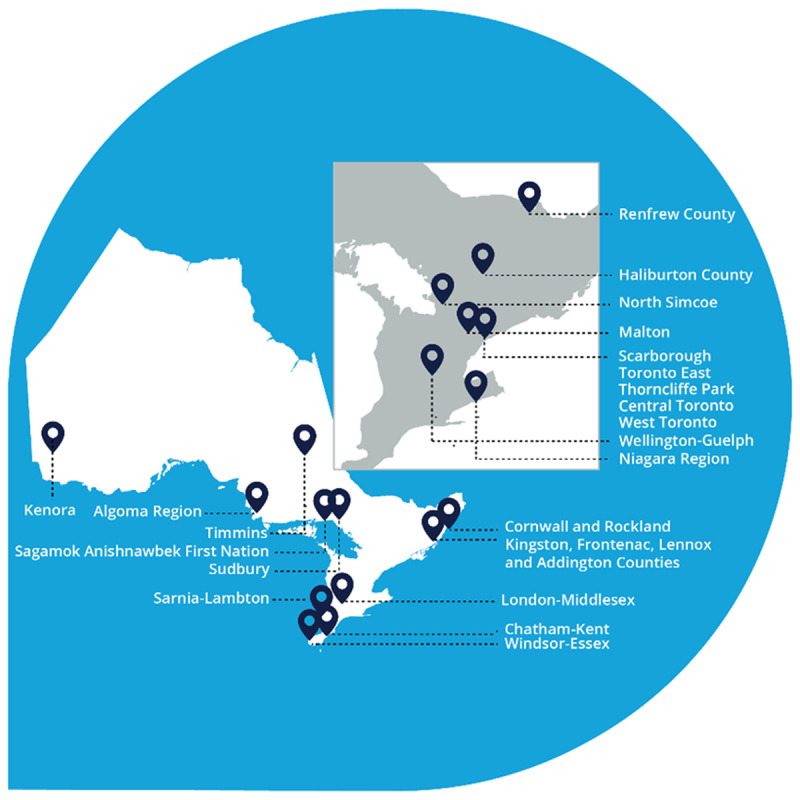
Youth Wellness Hubs Ontario sites.

In Canada, Youth Wellness Hubs Ontario (YWHO) is one of the most established of ten provincial IYS networks. YWHO co-leads a Federation of IYS Networks across the country that is building a pan-Canadian vision of improved mental health services and outcomes for youth through a learning health systems framework [17]. In Ontario, YWHO is a network of 22 (Figure 1) IYS networks, operating **31 hubs** where young people ages 12 to 25 years (thereby including access to transitional age youth) have walk-in access to youth-centered, community-based mental health and wellness services informed by youth, family members and service providers. YWHO is funded by Ontario’s Ministry of Health as well as philanthropic organizations who have played a key role in supporting and advocating for investment in youth mental health system transformation. Locally, YWHO sites also fundraise and seek funding from other sources to support the work in their community.

YWHO sites across the province are supported by a Provincial Office(PO) (often referred to as ‘backbone supports’) of implementation specialists, evaluators, knowledge brokers, equity specialists, communication experts, researchers, clinical staff, administrators, and senior leaders hosted by a large Ontario mental health and addiction hospital [10]. YWHO officially launched in 2017 with a 3-year demonstration phase involving ten sites. In 2020, annualized base funding was announced for the initial demonstration sites and in 2021 four additional sites were identified for funding. Presently, there are 22 networks across the province (see Figure 1).

YWHO sites are provincially-consistent in core features and locally led and adapted to offer and connect to a range of evidence-based, and developmentally appropriate services such as mental health care, substance use, primary health care, education, employment, housing, peer support, family support and care navigation in youth-friendly spaces. Services are expected to be integrated and co-located with a common consent form and shared processes and communication tools so that youth experience a seamless and less fragmented service experience. YWHO has also implemented standardized measures and a shared data platform province-wide that providers from all sectors are expected to use for service provision. Data from this platform are extracted by the YWHO PO evaluators and provided back to network leads in the form of service utilization reports on a quarterly basis, or when requested. The values and commitments that underpin the YWHO model include 1) meaningful engagement; 2) access, equity and inclusion for diverse youth; 3) high visibility and stigma-free; 4) integration across sectors; 5) continuous learning and quality improvement; and 6) service approaches that are youth-centered, developmentally-appropriate and holistic [10] (see Table 1 for YWHO’s core components). A full description of YWHO’s values, commitments, and model are available in Henderson et al., 2022. Many of YWHO’s core components are in line with the recently launched global framework for youth mental health [18].

**Table 1:** Youth Wellness Hubs Ontario core components and descriptions.


CORE COMPONENTS	DESCRIPTION

Youth and Family Engagement	Empowers youth and families to make decisions about their care by embedding their voice at all levels

Integrated Governance	Strategic collaboration between youth and service provider network to manage resources and organize service delivery

Accessibility	A comprehensive array of services that reflect the diversity of youth’s goals and needs

Culturally Diverse	Services that respond to the health beliefs, practices, cultural, and linguistic needs of diverse youth

Integrated Service Delivery	Integration of community-based service through a single, youth-friendly access point

Measurement-based Care	Use of standardized measures and outcome evaluation to enhance services to individual youth and to improve care


## Evaluation Methods

### Study Context

From January to February 2021, network leads from the first ten YWHO sites (launched in 2017), participated in an evaluation of the YWHO initiative. Notably, the onset of the COVID-19 pandemic took place during this demonstration phase. The objectives of the evaluation included identifying: 1) barriers and facilitators to the implementation of the model; and 2) service delivery modifications resulting from the COVID-19 pandemic. The main focus of this study was to identify factors that would further inform improvements to the implementation, sustainability, and scaling of YWHO across the province.

### Ethics and Consent

This evaluation was approved by the Quality Projects Ethics Review (QPER) team at the Centre for Addiction and Mental Health (QPER Ref#2021_028). Participation in this evaluation was voluntary. Participants were able to refuse to participate, decline to answer any questions or withdraw from the data collection activities at any time with no consequences. No minors participated in this study. Informed consent was obtained from all participants.

### Study Design

At the end of the first three years of the YWHO initiative, an evaluation was carried out to identify the barriers and facilitators to the initial implementation of YWHO and service delivery modifications resulting from the COVID-19 pandemic across ten sites. This evaluation used a pre-formative developmental approach [19] to integrate feedback and learning to improve the YWHO initiative as it continues to develop and scale. Given the highly unique, complex and dynamic contexts and systems in which this program operates, traditional forms of evaluation were not adequate in themselves.

### Data Collection

Network leads (N = 10) from all ten initial sites were invited to participate in site-specific 90 minute virtual key informant interviews (KII). Network leads are organizational leaders who oversee and provide accountability for the integrated network of service providers at their YWHO site. Proctor et al.’s [20] conceptual framework for implementation outcomes helped inform the development of semi-structured interview guides. Network leads discussed their role in implementation and adoption of the YWHO initiative, including barriers, facilitators and any modifications to service delivery made in response to COVID-19 amongst other implementation-related questions. Verbatim notes were taken during the interviews by first author (NV). Key informant interview data was not identifiable by name.

### Data Analysis

Notes from all interviews were analyzed using a reflexive thematic approach [21]. First author (NV) reviewed notes in full length and created a code set using inductive reasoning [22]. This code set was applied by the second author (DC) to a random sample of interview data to establish agreement, diversity and/or divergence from the initial code set. No new codes were identified. Disagreements were negotiated towards consensus. Qualitative coding and analysis was completed using Microsoft Office Excel. Supporting quotations were identified for each of the code sets by authors (NV) and (DC), with all identifiers removed. Themes were refined with agreement from the team.

## Evaluation Findings

Several facilitators and barriers to the implementation of the YWHO model during the demonstration phase were shared during the evaluation, however the most commonly identified factors are presented in this paper (Tables 2 and 3). Sample quotations from the network lead interviews that best exemplify the themes are included in Table 2. Facilitators included the diversified funding model, YWHO backbone supports, clear hub processes, strong community partnerships, and organizational support and dedicated staff. Most commonly identified barriers included staffing and financial resources, lack of integration of the shared data collection platform, inconsistent implementation of measurement-based care, partnership challenges, difficulties with integrated service delivery, and a lack of branding and communication support.

**Table 2:** Most common themes and sample quotations related to the facilitators of the implementation of YWHO.


FACILITATOR THEME	SAMPLE QUOTATIONS FROM NETWORK LEADS

1. Diversified funding model	*“We didn’t have infrastructure at the lead agency so [philanthropic funding] helped out with renovation dollars.”* *“At the beginning of COVID-19, the YWHO backbone gave us emergency funding to get the equipment we needed to provide services virtually. We got laptops, cameras for virtual groups, this was very helpful for us to move forward.”*

2. YWHO Provincial Office supports	*“The backbone team has been super helpful – without our backbone – I wouldn’t have even thought of it [implementation considerations].”* *“When we look for supports and need help, the response is do what works for you. The flexibility to contextualize is helpful, to do what works for our program, this has been helpful as well.”*

3. Strong community partnerships	*“Strong core partnerships – we got everyone to the table and identify what processes we can put in place quickly to reduce gaps in service offerings – did that quickly – like a week turnover.”* *“Representation on governance table from all sectors like [provincial enforcement agency] and child services – helped bring the conversation forward.”*

4. Organizational support and dedicated staff	*“Our operations table has really bought into the project – they are committed to making sure the project is successful, so we can give community and youth the best service. We brainstorm together, they enjoy trainings and they volunteer for working groups. Great buy-in from the frontline staff.”* *“Great leadership at core team and within partners and other agencies.”* *“Openness to adapt from the whole team*, *they accept that we need to do things differently and not everything will work the first time.”*

5. Clear hub processes	*“Core components laid out like that as a model for what we are trying to do – gave universal vision for us to focus on.”* *“There was an ongoing network leads meeting – direct communication with [leadership] and help understand the vision/thinking of the model. Hear what other sites are doing and how they are tackling issues.”*


**Table 3:** Most common themes and sample quotations related to the barriers of the implementation of YWHO during the demonstration phase.


BARRIER THEME	SAMPLE QUOTATIONS FROM NETWORK LEADS

1. Staffing and financial resources	*“Availability of service providers/clinicians is difficult in rural/remote contexts.”* *“Role of primary care isn’t as fulsome as it could be because for nurse practitioner they need a clinic room, neither of our sites have that. No [access to] full water or full bed, we say it’s primary care but it is quite limited.”* *“We don’t have funds for power buttons on bathrooms and elevator. It was hard to find commercial space, spent a year and a half trying to find physical space. Three year funding model was a barrier because we couldn’t sign a 10 year lease.”*

2. Implementation challenges of the shared data collection platform and measurement-based care	*“[Data collection platform] doesn’t allow for fulsome shared documentation, this prevents full integration, leads to double documentation, double consents, double registration.”* *“Speaking on the data piece, collecting the data virtually has been a challenge, so emailing forms ahead of time*

3. Partnership challenges	*“Partnerships are wonderful but partnerships should change as needs of communities change and that’s hard… resources are required to evaluate partnerships”* *“Joint training is difficult, all providers are in-kind, and each one has multiple service providers that circle through – no core team [during demonstration phase]. This makes it difficult to get them all there in one place, this takes away from service delivery. Ten hours of training for those with 3 hours at the hub doesn’t make sense.”*

4. Difficulties with integrated service delivery	*“At systems level each of our agencies need certain things. [Other service provider] only serves 16–20 year olds, it didn’t even cross our minds, so we serve 12–25 and now we have a list of different age ranges and who we can send all the different intake processes and different questions. How do I count numbers? This gets in the way of delivering.”* *“Need for monthly getting together of setting goals and benchmarks for clinical goals and making sure all sites are doing it. For example standardized information on harm reduction for youth, otherwise hamburgers taste different at each [restaurant].”*

5. Branding and communications challenges	*“The fact that we weren’t already there for them to know our services, getting the word out and services known is one of the biggest challenges.”* *“Not having a communications specialist helping us with social media… this promotion needs to be constantly there, youth awareness of our services need to be better.”*


The COVID-19 pandemic was arguably the most significant barrier (Table 4) to the implementation of the YWHO model, especially as many sites opened doors immediately before the pandemic was declared. Several modifications to service delivery were necessary. Sites without longstanding physical presence in the community struggled with YWHO brand recognition by youth and therefore getting youth in the doors. Sites that were not previously active on social media were propelled to build their online presence quickly to raise awareness about hub services and used social media as a platform for service delivery. Communication via social media also became increasingly important with the loss of a physical access point during lockdown. Sites had to invest already constrained resources on social media activity/virtual platforms. Sites also offered virtual presentations on hub services at schools to increase uptake of hub services and increase youth’s familiarity with hub staff by conducting outreach and programming on social media/virtual platforms. The largest modification to service delivery during the COVID-19 pandemic was the pivot to virtual service delivery, in some cases exclusively. Sites had to work quickly to sort out privacy and security issues, determine consent processes, identify videoconferencing platforms and establish a help-desk type phone line amongst a multitude of other tasks. Results are expanded upon in the discussion section.

**Table 4:** COVID-19 Pandemic and modifications to service delivery.


COVID-19 THEME	SAMPLE QUOTATIONS FROM NETWORK LEADS

1. Building online presence (social media for marketing and program delivery)	*“Increased social media presence. Communication and marketing became really important because no physical access point*, *we put time and funds into that– silver lining.”* *“Then how to be innovative in reaching youth virtually – this is challenging, not being in person and promote something virtually that was originally supposed to be in person”*

2. Pivot to virtual service delivery	*“Had to get a platform – we have [videoconferencing platform] and had to figure out which platform to use for service. Using a phone-line, a number that youth can be familiar with, kind of like a helpline.”* *“Virtual counselling, we moved in three days when our original plan was to implement in 2022. Thinking about phone counselling was way ahead in the future but we did it so quickly.”* *“Some youth couldn’t leave home due to depression and other things, but now we can bring programming to them, which we couldn’t do before, COVID has changed the way we think and how we do social work.”*

3. Increased community outreach and advocacy	*“Trying to address the Social Determinants of Health. Other folks in community are raising awareness of the internet issue, as a basic human right and youth have been engaged to speak to that by attending council meetings and speaking to press.”* *“Outreach, we picked places in the community. We offer gift cards, masks, snacks and see youth that can’t travel. Have some sort of connection and just reinforce the services. We let them know about what’s happening virtually, trying to get them to come back to us. We give them phone number and stuff and get relationship that way.”*


## Discussion

The main objective of this evaluation was to assess the barriers and facilitators of the implementation of YWHO during the demonstration phase from the perspectives of network leads. Insights have been generalized in a lessons learned format, which is intended to benefit those seeking to support youth in their communities to design and implement services that improve youth mental health and overall well-being.

### Facilitators to implementation of the YWHO model

This evaluation identified several facilitators including a flexible and diversified funding model, provincial backbone supports, clear hub processes, strong community partnerships, and organizational support and dedicated staff. Receipt of provincial funding for YWHO supported its credibility and helped with staff buy-in and commitment towards integrated care which has been identified as a key facilitator in providing youth care [15,23]. Access to philanthropic funding enabled sites to make much needed renovations and emergency funding provided at the start of COVID-19 and was essential for purchasing equipment to implement the unexpected pivot to virtual service delivery. Diversified funding streams that allow for adequate resourcing and staffing will enhance service delivery and fidelity to the YWHO model similar to findings from other studies [15].

Provincial Office backbone supports were identified as essential facilitators to the implementation of the model. Key supports included administration and project management; fundraising; data collection, analysis and dissemination; various coaching supports (e.g. health equity, clinical content, youth engagement, Indigenous content etc.); connection to sector resources; and partnership building. These backbone functions have been most extensively described in the collective impact literature [24] as essential to the success of any multi-sector partnership. Regularly held provincial network leads meetings and direct lines of communication with YWHO leadership were also identified as critical for staying connected across hubs and understanding how other sites tackled issues. As described in previous studies [15,25], clear and consistent communication across integrated care team members and leadership is essential to ensure that on-the-ground operations proceed smoothly.

Clear hub processes as described in several studies highlighted in Nooteboom’s systematic review of facilitators and barriers for professionals [15] including establishing clear referrals to external organizations, service pathways, and collective service planning among partners were significant processes that impacted integrated service delivery at YWHO sites. Shared calendars and promotion of services through social media helped with scheduling services and to increase youth attendance. Existing strong formal partnerships with community agencies that support youth specific initiatives who were willing to provide in-kind services enabled the implementation of the YWHO model as highlighted in previous research [9,23]. Representation of partners on governance tables from diverse sectors helped to keep the work moving. Meeting with partners to identify processes that can be implemented to reduce gaps in service offerings was beneficial as well.

Finally, organizational support and dedicated staff, including strong leadership from network lead organizations was instrumental in implementing the model. A network lead that was available, engaged and accessible to site partners, who provided spaces for service providers to co-locate, collaborate and deliver services was essential for integration, as outlined previously [23]. Establishment of operations tables at sites that meet regularly, troubleshoot efficiently, and share training and programming updates with each other was instrumental. Committed working groups composed mostly of service providers participated in trainings and brainstormed together to improve services for youth in the community. Engaging youth in service design and delivery was a key consideration. These dedicated staff support the YWHO vision, are passionate, dedicated and essential for the success of the YWHO model.

### Barriers to implementation of the YWHO model

Common barriers included staffing and financial resources, challenges with implementation of the shared data collection platform and measurement-based care, partnership challenges, difficulties with integrated service delivery, and a lack of branding and communications support. For the demonstration phase of the ten YWHO sites, funding was available for three years (2018–2020). This posed several challenges: difficulties hiring staff on contracts, signing leases for long-term hub spaces, and out-sourcing additional expertise (e.g. Information Technology, marketing etc.). Budgetary challenges when establishing IYS is a common barrier that has been documented previously [12,23]. Funding was also limited for infrastructure changes that would increase physical accessibility, remove transportation barriers and difficulties with accessing spaces for providing appropriate primary care services (e.g. examination rooms). Adequate staffing was also challenging, especially in rural/remote areas where few in-kind service providers were available and high-turnover of health services staff in general. With the announcement of new annualized base provincial funding in 2020, the first 10 sites received funding for five Youth Wellness Team positions (mental health & substance use clinician, nurse practitioner, peer support worker, care navigator, and intake coordinator). These distinct roles will improve integrated service delivery [15].

Many sites initially struggled with the integration of measurement-based care (MBC) in practice. Common reasons included: competing priorities and limited session time; clinician perception of standardized tools; use of different screeners or other pre-existing data collection requirements; need for additional training; and requirements for data entry into more than one electronic record system. Barriers of this nature have been well documented elsewhere [26].These barriers initially contributed to inconsistent use of standardized measures and reporting of outcomes in some sites. Changes have since been implemented that have helped to improve the use of MBC, as well as data collection within YWHO sites. These include, data platform improvements, tailored implementation supports, increased training and community of practice supports, data quality reports, and the creation and upkeep of the YWHO Knowledge Base – an online resource that hosts content about the implementation of MBC, among other resources. Multifaceted change management processes are needed to target local barriers to data collection and implementation of MBC [26].

With respect to partner collaboration, some participants mentioned an interest in completing readiness assessments with partners in order to ensure partners are able to take ownership of service delivery at the hub and understand the YWHO model. These processes have been identified in prior work on forming and sustaining partnerships in IYS [27]. Joint training was also identified as a challenge from a capacity perspective as many providers deliver in-kind services and it is difficult to gather staff in one place at one time without impacting service delivery. Regular evaluation of and change in partnerships as community needs shift was also identified as being difficult but necessary. However, participants noted it is challenging to monitor the output and commitment of partners because of the time and resources required to do this.

Integrated service delivery is a key core component of YWHO and one that participants described as difficult to implement. Challenges at some sites included not enough integrated team meetings with adequate communication mechanisms in place. Strong and effective leadership is needed from network leads to ensure diverse representation from staff, youth, and partners and that decisions are communicated regularly in a transparent manner [15,23,27]. YWHO brand adoption is another core component that participants experienced challenges implementing. Sites needed to boost their online presence and visibility in general, especially those sites that did not have a physical hub before the pandemic. However, during the demonstration phase limited central funding for marketing and communications was available. Since that time, YWHO has addressed this need by hiring a senior communications person to support marketing and communications across the network.

### COVID-19 Pandemic and modifications to service delivery

The COVID-19 pandemic shifted the implementation of the YWHO model in multiple ways. Like many providers who deliver services to children and youth, hubs have had to boost their online and social media presence in order to build awareness of hub services. However, the largest modification to service delivery was the pivot to almost exclusive virtual service delivery during the first year of the pandemic. This mode of health care delivery was a double-edged sword for youth as described elsewhere [28,29,30], especially for those who live in rural/remote communities with low bandwidth or no access to electronics. In these cases, sites worked with partners to identify an equitable process to distribute cellphones or identify safe places in the community where youth can access virtual services (e.g. use of guidance counsellor’s offices in schools). The benefits of virtual service delivery include the removal of transportation as a barrier by bringing programming right to their doors, as well as providing increased access to specialist services, especially in rural/remote settings. Texting support with peer support workers was also introduced by some sites, this is often the first point of contact before youth are transitioned to other hub team members as needed. Despite challenges with virtual service delivery, most sites plan to continue offering virtual services consistent with findings from other studies evaluating rapid implementation of virtual service delivery for youth during COVID-19[30].

Finally, hubs increased their outreach and advocacy work to address community issues that had been exacerbated because of the pandemic. Sites identified key places in the community to conduct pop-ups where they handed out gift cards, masks, snacks and saw youth who were unable to travel to the hub. The aim was to strengthen relationships and increase awareness of hub services both over the telephone and virtual offerings. The pandemic has forced sites to reimagine service provision for youth.

### Lessons Learned

The major lessons learned from this evaluation can be organized as insights under two of the YWHO core components.

Based on the lessons learned from the YWHO evaluation, improvement initiatives continue in several areas: increasing communication touchpoints and expansion of a centralized role to provide consolidated communications for YWHO; increasing collection of sociodemographic data and better understanding of youth in site region; improving accessibility of space and services (hours of operation, visibility, cultural spaces, service offerings in numerous languages, diverse staff, culturally appropriate tools and healing practices); critical reflection of partnerships; and, developing a shared understanding of integrated service delivery across all partners.

**Table:** 


CORE COMPONENTS	LESSONS LEARNED

**1. Integrated Service Delivery**	**Optimize integrated service delivery by:** Ensuring all hub providers have a thorough understanding of the diverse services offered at the hub, including familiarity with different service pathways; Partnering with schools and other community organizations close to where youth live to provide private spaces to access virtual services, where bandwidth/technology are additional barriers to accessing services; Physically co-locating multiple service providers in the same space; Offering virtual service delivery to increase access to services for rural/remote youth and youth who are unable to leave their homes, as well as connection to specialists like psychiatrists. Documenting clear hub processes, including clear governance structures, signed Memorandum of Understandings and service agreements; Holding regular team meetings with all staff to prevent communication breakdowns; Providing access to Implementation Specialists, including for support with project management, administrative support, and troubleshooting implementation challenges; Offering Knowledge Broker support through the creation of evidence briefs, content for training, community of practice spaces, and youth engagement materials; Establishing hub operations tables to troubleshoot and share training and programming updates; Preparedness to troubleshoot scheduling issues that can result from partners having different service formats (e.g. mix of in-person and virtual services); Sharing calendars and promoting services through social media; Having a shared definition of integrated service delivery and alignment of integration goals by network leads and partners; Completing centralized training in order for all providers to have a shared understanding of the model; Implementing a common consent form; Ensuring an online and social media presence; Offering drop-in and scheduled services

**2. Measurement-based Care**	**Success with implementing MBC can be achieved by:** Access to a Clinical Practice Leader who can support implementation and interpretation of various standardized measures; Access to Evaluators who can support data collection, analysis, and reporting related to the integrated data collection platform; Implementing a measurement-based care data platform that better integrates with existing systems and can serve broader documentation functions Implementing change management processes to mitigate challenges related to use of a minimum common set of measures for measurement-based care and review of responses with youth to make care decisions.; Incorporating more culturally relevant tools into the common measurement set to increase relevance to some groups, including Indigenous youth.


## Limitations

One of the limitations of this work is that the evaluation was carried out by the YWHO Provincial Office and therefore there is the potential that key informants’ responses may have been impacted by social desirability [31]. To reduce the impact of social desirability, however, all interviews were conducted by a new staff member who had not yet provided implementation or site support at the time. As this model was implemented in Canada, a high-income nation, generalizability of results may be more limited in more resource-stressed countries. Another limitation is that these findings are from the perspectives of network leads and may not be representative of other YWHO partners such as service providers, youth, and families. Future evaluations should report on perspectives from direct service providers as well as youth and families accessing YWHO services.

## Conclusion

Implementation of IYS is highly collaborative and quite complex. As interest in these models increase, so does the need for knowledge related to optimal implementation. Learnings can inform how community leaders, including youth, design and implement services to improve youth mental health and overall well-being. Future studies should assess youth experience in accessing and receiving services from IYS.
